# Three-Dimensional Mapping of Soil Chemical Characteristics at Micrometric Scale by Combining 2D SEM-EDX Data and 3D X-Ray CT Images

**DOI:** 10.1371/journal.pone.0137205

**Published:** 2015-09-15

**Authors:** Simona Hapca, Philippe C. Baveye, Clare Wilson, Richard Murray Lark, Wilfred Otten

**Affiliations:** 1 School of Science, Engineering and Technology, Abertay University, 40 Bell street, Dundee DD1 1HG, United Kingdom; 2 Laboratory of Soil and Water Engineering, Department of Civil and Environmental Engineering, Rensselaer Polytechnic Institute, Troy, New York, United States of America; 3 Unité EcoSYS, AgroParisTech, Université Paris-Saclay, Avenue Lucien Brétignières, F-78850 Thiverval-Grignon, France; 4 School of Natural Sciences, University of Stirling, Stirling FK9 4LA, United Kingdom; 5 British Geological Survey, Nottingham NG12 5GG, United Kingdom; NERC Centre for Ecology & Hydrology, UNITED KINGDOM

## Abstract

There is currently a significant need to improve our understanding of the factors that control a number of critical soil processes by integrating physical, chemical and biological measurements on soils at microscopic scales to help produce 3D maps of the related properties. Because of technological limitations, most chemical and biological measurements can be carried out only on exposed soil surfaces or 2-dimensional cuts through soil samples. Methods need to be developed to produce 3D maps of soil properties based on spatial sequences of 2D maps. In this general context, the objective of the research described here was to develop a method to generate 3D maps of soil chemical properties at the microscale by combining 2D SEM-EDX data with 3D X-ray computed tomography images. A statistical approach using the regression tree method and ordinary kriging applied to the residuals was developed and applied to predict the 3D spatial distribution of carbon, silicon, iron, and oxygen at the microscale. The spatial correlation between the X-ray grayscale intensities and the chemical maps made it possible to use a regression-tree model as an initial step to predict the 3D chemical composition. For chemical elements, e.g., iron, that are sparsely distributed in a soil sample, the regression-tree model provides a good prediction, explaining as much as 90% of the variability in some of the data. However, for chemical elements that are more homogenously distributed, such as carbon, silicon, or oxygen, the additional kriging of the regression tree residuals improved significantly the prediction with an increase in the R^2^ value from 0.221 to 0.324 for carbon, 0.312 to 0.423 for silicon, and 0.218 to 0.374 for oxygen, respectively. The present research develops for the first time an integrated experimental and theoretical framework, which combines geostatistical methods with imaging techniques to unveil the 3-D chemical structure of soil at very fine scales. The methodology presented in this study can be easily adapted and applied to other types of data such as bacterial or fungal population densities for the 3D characterization of microbial distribution.

## Introduction

Many soil properties and functions emerge from interactions of physical, chemical and biological processes at microscopic scales [1], but for many decades, measurements at this scale have remained largely unattainable. However, tremendous technological progress over the last decade has allowed soil scientists to observe and quantify the microscopic heterogeneity of soils in ways heretofore unmanageable.

Researchers now have routine access to X-ray computed tomography systems, either synchrotron-based or table-top, which, thanks to recent methodological improvements (e.g., [2–9]), provide increasingly reliable information about the geometry of pores and solids in soils at resolutions as small as 0.5 μm. Concomitant progress in near-edge X-ray spectromicroscopy (NEXAFS), synchrotron X-ray absorption spectroscopy, and synchrotron-based microfluorescence spectroscopy of thin sections of soils has led to observations of sharp spatial heterogeneity in the chemical make-up of soil organic matter over minute distances, respectively of the order of nanometers to micrometers [10–12] and in the accumulation of trace metals [13–17]. Significant advances related to biological markers also allow specific bacteria to be identified in soils and their spatial distribution at micrometric scales to be determined in thin sections [18,19]. More recently, use of nanoscale secondary ion mass spectrometry, coupled with transmission electron microscopy, has made it possible to image, and assess spatially, the differential partitioning of NH_4_^+^ between plant roots and native soil microbial communities at the submicron scale [20].

For the first time, the availability of these different measurement techniques, operating at a spatial scale commensurate with that of bacteria and clay particles, enables researchers to try to understand in great detail how soils function at that scale. However, to do so satisfactorily, a key requirement is to be able to fully integrate existing measurements, i.e., ideally to determine at the same resolution and at the same locations how physical, chemical, and biological soil parameters vary spatially and over time. This superposition of different measurements is currently feasible in the 2-dimensional spaces corresponding physically either to thin sections of resin-impregnated, solidified soil blocks, or simply to cross-sectional cuts through soil aggregates or small cores. Unfortunately, this information on physical, chemical, and microbiological parameters determined on 2D planes within soil samples has limitations in terms of unravelling soil processes. Evidence has abounded for some time that many aspects of the behaviour of soils, including their transmission of liquid phases and solutes, can be described accurately only on the basis of 3-dimensional information [21–27]. For example, the degree of connectivity or tortuosity of the pore space in 2-dimensional cuts through a soil is generally different than in 3-dimensions, and only the full 3-D connectivity is relevant when one attempts to predict the hydraulic conductivity or dispersion coefficient of a given soil sample, or biomass spread (e.g., [28]). In a similar way, in order to describe realistically how microscale heterogeneity affects processes that involve (bio)chemical reactions, 3-dimensional spatial data about the chemical make-up of soils, or at least about the distribution of targeted compounds, is necessary.

Micro-spectroscopic techniques, which will allow the 3-dimensional, quantitative mapping of soil elemental composition at micrometric resolution, are currently not available. Significant progress has been made recently in this direction, using synchrotron-based μXRF, in small-sized samples of materials that are comparatively far less heterogeneous chemically than soils typically are [29,30]. To apply 3-D μXRF to soils, several technological hurdles need to be overcome; in particular the attenuation of fluorescence X-rays inside the soil samples. Another approach, adopted by a number of researchers in the last few years (e.g., [31,32]), is to map specific elements in 3D in soils by taking advantage of the fact that their X-ray absorption differs markedly below and above a definite energy level (K-edge). However, this method is not suitable for all compounds, and it requires monochromatic X-rays, which for the time being, are available only at a small number of synchrotron facilities around the world.

Until direct 3D mapping of the chemical properties of soils becomes technically feasible and more accessible, an alternative approach to obtain 3-dimensional chemical information in soils is to perform multiple cuts through soil samples, analyse in turn the (bio)chemical make-up of each exposed surface and the spatial distribution of biological populations within the soil, then, using interpolation technique, generate a 3-D picture from the data associated with the various surfaces. This procedure is routinely used for biological samples, such as human tissues, in which the serial removal of layers can be carried out easily, either by using a microtome or via ion-beam ablation. However, in mineral soils, the frequent presence of dense constituents reduces the range of techniques that can be used to cut or scrape away successive layers with minimal disturbance. Furthermore, particularly when operating microtomes, the presence of constituents with markedly different densities often causes blades to deviate from their set course, so that eventually the exposed surfaces are not perfectly flat. Because of that, the correspondence of 2-D chemical or microbiological maps with the physical information obtained via computed tomography is likely to be poor, unless artefacts generated during soil cutting are accounted for.

Therefore, a first step in any attempt to simultaneously evaluate in 3D the physical, chemical, and biological characteristics of soil samples is to find a way to correct for any distortion that may occur when cutting or grinding down soil samples to successively expose surfaces on which 2-D chemical mapping is carried out, and to geo-reference these 2-D maps within the geometry of the soil solid phase, determined via X-ray computed tomography. A practical, automated procedure to accomplish these tasks has recently been developed by Hapca et al. [33]. This procedure involves three successive steps, namely the reconstitution of the physical structure of a given soil layer surface, the alignment of the chemical maps with the reconstituted soil surface image, and finally the 3D alignment of the 2D chemical maps with the internal structure of the soil cube. Visual comparison of elemental maps and of the reconstituted CT images of the layer surfaces suggested a good correspondence between them, which was supported by a correlation analysis of the different surfaces and elements considered. This good agreement suggests that it is now possible to proceed through interpolating statistically between successive geo-referenced 2D planes, and predict in 3D various (bio)chemical and microbiological characteristics of soils.

The key objective of the present article is to develop a statistical method to produce 3D maps of the spatial distribution of chemical elements in soils at microscopic scales based on the 3D characterisation of the soil structure provided by X-ray CT images and the 2D spatial distribution of chemical elements obtained by SEM-EDX. Application of this method to a particular soil from Shetland illustrates what results can be expected.

## Materials and Methods

### 2.1. Sample preparation and procedure

The sample was obtained from a farm in Papa Stour, Shetland, UK (60°19’44”N 1°40’57”W) with the permission of the land owner. The soil used is a freely-drained brown calcareous soil of the Fraserburgh Association (Soil Survey of Scotland, 1981) that has developed in wind-blown quartz sand, producing a soil with a sandy loam texture. The soil had been cultivated using traditional methods until 1939, and since then the field has been used as permanent pasture. As an old arable soil, the Ap horizon was probably relatively well homogenised, but since the soil has been under grass for the last 70 years, the architecture of the soil is now well developed and differentiated in depth. The soil organic matter content is high, at 13%, and remnants of past manuring are frequent, including fragments of bone, charcoal, shell and pottery [34] that add to the heterogeneity of the soil chemical matrix.

An undisturbed block (7 × 6 × 5 cm) of soil was cut from the profile face of a 60 × 60 cm wide test pit at a depth of 8–15 cm using a Kubiena tin. The undisturbed sample was dried in acetone vapour, and then impregnated under vacuum with epoxy resin using procedures described by Wilson et al. [34]. The resulting block was cut to produce a 1 × 1 × 1.3 cm soil cuboid from its centre.

### 2.2. X-ray computed micro-tomography

The 3-dimensional pore space of the soil was visualised with an HMX micro-tomography system (Nikon Metrology, Tring, Herts, UK). The whole cuboid was scanned at a resolution of 11.9 μm, and an energy of 130 kV, a current of 75 μA, 3010 angular projections, and a 0.25 mm aluminium filter to reduce beam hardening. To obtain images at the same resolution as the chemical maps generated subsequently via SEM-EDX, the data were reconstructed using CT-Pro v. 2.0 (Nikon Metrology, Tring, Herts, UK) at a 15.8 μm resolution. VGStudio Max v. 2.0 was used to convert volumetric images into a 16-bit grayscale image stack. Then Matlab v.8.2 was used to map the 16-bit grayscale image stack into an 8-bit bmp image stack following the outlier correction procedure described in Houston et al. [35,36]. In total, 1070 2D greyscale images were obtained for the whole soil cuboid.

### 2.3. Soil sectioning and SEM-EDX scanning

**Fig 1:**
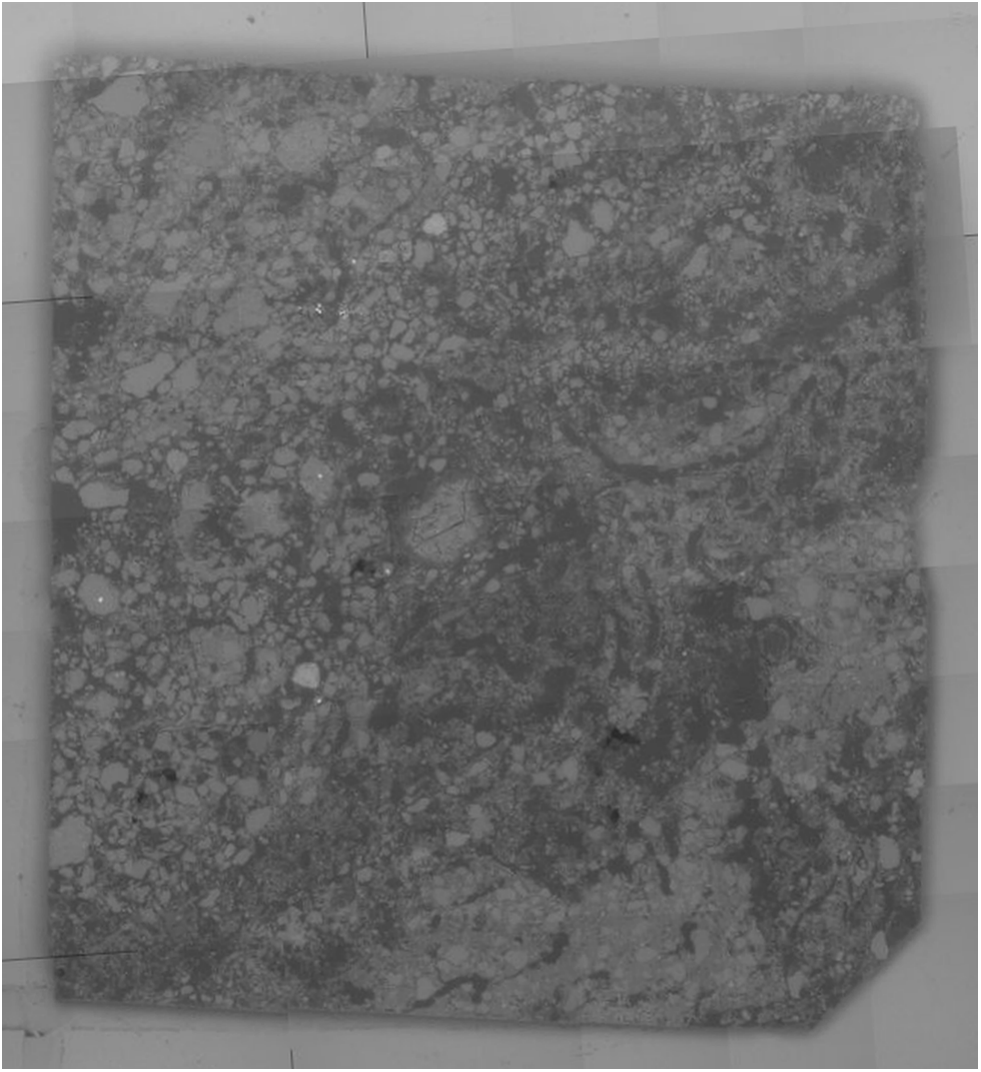
Chemical mapping by SEM-EDX. Example of soil slice approx. 250μm in thickness, which was scanned by SEM-EDX for chemical mapping.

After the cuboid was scanned, it was cut to produce parallel slices with a thickness as close as feasible to 250 μm (Fig 1). Due to the blade width (kerf) a layer of approx. 790 μm in thickness was lost during the cutting process. The top five slices, representing the upper 0.45 cm of the cube, were scanned on both sides by energy-dispersive X-ray spectroscopy (SEM-EDX) to identify chemical components. The SEM-EDX scanning was done under low vacuum conditions (60 Pa) to protect against surface charging and to alleviate the need to apply a conductive coating. A Zeiss EVO-MA15 SEM fitted with an Oxford Instruments INCA Max 80 mm EDX detector was used to quantify and map elemental spatial distributions across soil slice surfaces. The INCA Automate and Mapping software was used for the data collection and initial data and image processing. The SEM-EDX analyses were carried out under strictly standardised operating conditions of 50 μA gun current, 2.525 A filament current, 20 kV accelerating voltage, and 8.5 mm working distance to achieve an acquisition rate of 10 kcps. An external Co standard was analysed at the beginning and end of each analytical run to ensure stable beam conditions, and X-ray count rates were maintained throughout data acquisition (5% deviation tolerance). To provide coverage of the 1 cm^2^ slice surfaces, a series of 70 SEM-EDX scans were obtained from each face using a magnification of × 71 and a field of view of 3993 μm × 2995 μm. A step move of 2 mm between sequential images in both the X and Y dimensions was made to give a 30% minimum overlap between fields of view for subsequent image mosaicing. Each individual scan image had an ultimate pixel size of 7.8 μm and a total X-ray count of >5 million.

The SEM backscattered electron images and associated X-ray maps were mosaiced using INCA Automate (INCA 17.1 software) to produce a SEM-BSE image and a series of element maps for each slice face with a pixel size of 15.8 μm. For each mapped element (Al, Si, O, C, Fe, K, Ti), characteristic X-ray counts from each image pixel were exported and saved as an excel file.

### 2.4 Alignment of the 2D SEM-EDX chemical maps with the 3D X-ray CT volume

The method developed by Hapca et al. [33] was applied to each of the ten SEM-EDX chemical maps (two sides per slice) to locate the corresponding 2D plane within the 3D volume corresponding to the X-ray CT image. Given that the 2D surfaces were not perfect squares (Fig 1) a rectangular region of interest of 750 × 800 pixels was cropped from each of the original 2D SEM-EDX map. At the same time, the 3D X-ray CT data of the physical structure was represented as a sequence of 2D grayscale images, on each of which a region of interest of 830 × 880 pixels being cropped and used for alignment. The alignment method (Hapca et al., 2011) [33] consists of searching systematically through the 2D grayscale image stack of the physical data, and reconstructing a rotated plane that correlates best, statistically, with the chemical maps of carbon, silicon, iron and oxygen simultaneously, this being done for each of the ten 2D surfaces on which SEM-EDX data were collected. The rotation of the plane was carried out with respect to the geometric centre of the layer, located at (*x*_0_, *y*_0_) = (15.8μm×*i*_0_, 15.8μm×*j*_0_), with *i*_0_ = 415, *j*_0_ = 440. After this rotation, the z coordinate of any point in the plane can be expressed via the following equation:

$$\begin{array}{l} z=a(x-x_{0})+b(y-y_{0})+z_{0}\\ \;=a(i-i_{0})\times 15.8\mu \text{m}+b(j-j_{0})\times 15.8\mu \text{m}+k_{0}\times 15.8\mu \text{m} \end{array}$$

For *a* = *b* = 0, the initial, un-rotated plane is recovered. If, for example, the plane is rotated first by an angle *φ* around the *x* axis and then by an angle *ψ* around *y* axis, then parameters *a* and *b* in the above equation correspond to *a* = tan(*ψ*) and *b* = tan(*φ*)/cos(*ψ*).

For the data in this study, the search space for parameters (*a*, *b*) was in steps of 0.001 within (−0.05, +0.05), corresponding to rotation angles *φ* and *ψ* varying between approximately −3^*o*^ to 3^*o*^. Although the equation above allows *z* to vary continuously, given the discrete nature of the scanned data, the *z* coordinate of each point in the rotated plane needs to be approximated by the integer that is closest to *z* / 15.8μm, producing a staircase-like geometry for the rotated plane. For each rotation parameters *a* and *b*, after reconstructing the 2D X-ray grayscale rotated plane, which is 830 × 880 pixels in size, another search needs to be performed within this plane to find and then crop a region of 750 × 800 pixels that correlates best with the chemical maps. The maps of carbon, silicon, iron and oxygen were used simultaneously in the alignment procedure by means of a cumulative absolute value of the correlation coefficient between the 2D reconstructed physical plane and each of the four 2D chemical maps. Optimum rotation and displacement parameters estimates were determined based on maximizing the cumulative correlation.

### 2.5 Statistical approach to predict the 3D chemical composition of soil by combining 2D SEM-EDX with 3D X-ray CT data

To obtain a 3D map of the chemical composition of soil, a regression tree statistical approach was developed together with a method of cross-validation to assess its performance.

#### 2.5.1 Regression tree and regression tree kriging

Regression kriging (RK) is a spatial interpolation technique that consists of first carrying out a regression of the dependent variable on auxiliary variables, and then kriging the regression residuals. In this respect RK approximates the empirical best-linear unbiased prediction (E-BLUP) which is also obtained by Universal Kriging (UK) when the regression is to account for a spatial trend and Kriging with External Drift (KED) corresponding to the use of additional covariates [37]. RK is only approximate because the estimate of the random model from regression residuals is biased, but this bias is small with large samples, and the proper estimation of the model by likelihood methods from very large samples is computationally demanding [38]. In the RK framework one may also use a regression component obtained by methods such as regression trees which do not fit into the linear mixed model framework [39].

If we denote by *Z*_*u*_ the spatial variable that we want to predict and by *Z*_*v*_ the auxiliary variable which is sampled more often than the first variable, then RK decomposes the variable *Z*_*u*_ into the sum:

$$Z_{u}(x)=f(Z_{v}(x))+\varepsilon (x),$$

where, *f*(*Z*_*v*_(*x*)) is a deterministic component and *ε*(*x*) is a stochastic component representing the residual error. In regression kriging, the deterministic part is fitted first using ordinary least squares, and then the residuals are estimated by a method of interpolation such as Ordinary Kriging (OK).

**Fig 2:**
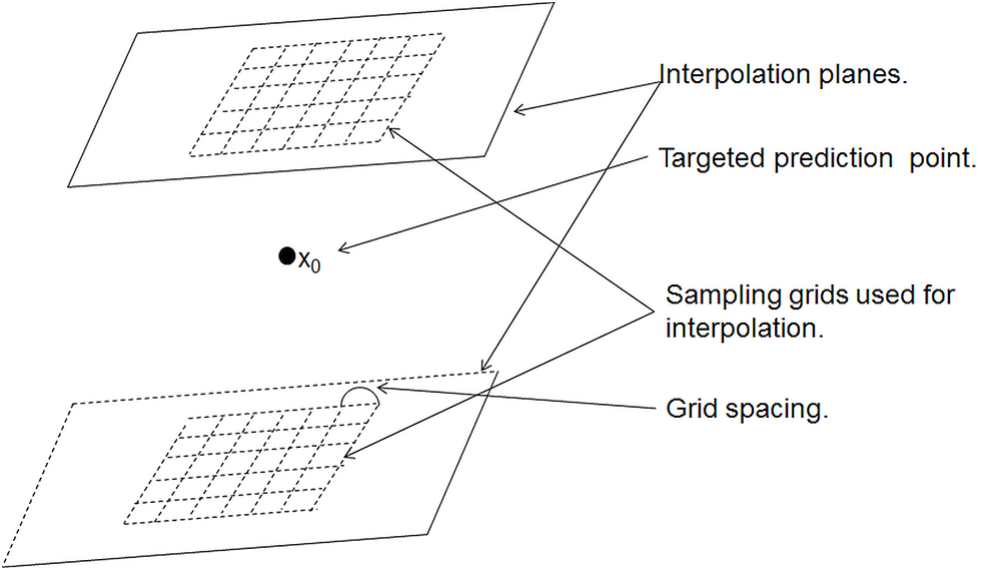
Illustration of the sampling grids used in kriging. Schematic representation of the interpolation layers and the corresponding sampling grids for the selection of the interpolation points. The actual distance between the top and bottom layers, on which the concentration of chemical elements is measured, depends on the cutting and polishing processes, and may be variable.

Regression kriging was preliminarily implemented and tested using two forms of the deterministic component, a simple linear regression and a regression tree, consisting of a linear combination of step functions as described in Hastie et al. [40]. However, due to the relatively poor predictive performance of the linear regression, only the regression tree method will be reported in this study. For any two layers from the 3D soil cube on which 2D chemical measurements are available (Fig 2), the regression tree kriging method consists first of regressing the chemical measurements (one chemical elements at a time) on these two layers against the corresponding 2D X-ray CT grayscale data (resulted from the alignment procedure above) by regression tree. Then residuals on these two layers are then calculated and interpolation by OK is used to estimate residuals for any point *x*_0_ within the 3D internal domain between the two interpolation layers. Based on these two approaches, the chemical element within the 3D internal domain can be predicted as follows:

$${\hat{z}}_{u}(x_{0})=\hat{T}(z_{v}(x_{0})),\;\text{for the regression tree method alone},$$

and

$${\hat{z}}_{u}(x_{0})=\hat{T}(z_{v}(x_{0}))+\hat{\varepsilon }(x_{0}),\;\text{for the regression tree kriging method},$$

where $\hat{T}$ is the fitted value according to the regression tree, $\hat{\varepsilon }$ is the residual predicted by OK, and *x*_0_ is a point within the 3D internal domain between the two interpolation layers, for which prediction is sought. The regression tree was fitted using an existing procedure available in Matlab v.8.2 under the Statistics toolbox. The OK procedure for residual prediction was implemented by the authors in MATLAB v.8.2 following the theory described by Webster and Oliver [41], which consists of a linear interpolation that is optimal in the sense that observed values within a neighbourhood are linearly combined to form a prediction at the site of interest in such a way as to produce an unbiased prediction which minimizes the error variance.

#### 2.5.2 Modelling the variogram and selection of the optimum sampling grid for regression kriging

Due to computational constrains, in this study, we assumed an isotropic variogram model. Based on the profile of the experimental variogram, exponential and spherical models or a combination of those with or without nugget were fitted to the residual data obtained from the upper and lower layers. Variogram model selection was carried out based on the Akaike Information Criterion (AIC) as described in Webster and Oliver [41], and the model that provided the best fit was used to obtain the OK prediction of the residuals.

In addition to selecting the right variogram model and solving the kriging system, a key step in kriging consists of choosing the points *x*_1_, *x*_2_,⋯, *x*_*n*_, in the neighbourhood of the prediction point *x*_0_, to be used in the interpolation [41]. In studies using image data, such as the one reported here, where there are very large numbers of observations, it is necessary to consider computational constraints and to select a restricted subset of observations from which to krige. Conventionally, when data are dense and regularly sampled in all dimensions, one may select all neighbouring observations within a distance of the target point equal to the range of correlation of the variable [41]. In this study the data are available in two layers 65 voxels apart with the target point for kriging in between them. The 2*N* observations closest to the target point form two clusters, each of *N* observations, one on each layer, are normally used in the kriging interpolation. The observations within each cluster are strongly correlated with each other, so this is not necessarily the best set of observations from which to predict. Given the variogram, the kriging variance for a prediction can be computed from the spatial distribution of observations relative to the target point. In this study the kriging variance was computed for square grids of *N* = *n* ×*n* observations, selected from each of the two layers and centred about the projection of the target point onto the two layers (Fig 2). The grid spacing took values from 1 to 20 voxel units. The kriging variance was plotted against the grid spacing to find the array of fixed size which minimized the kriging variance. This configuration of observations was then used for all kriging.

#### 2.5.3 Model implementation and cross-validation

**Fig 3:**
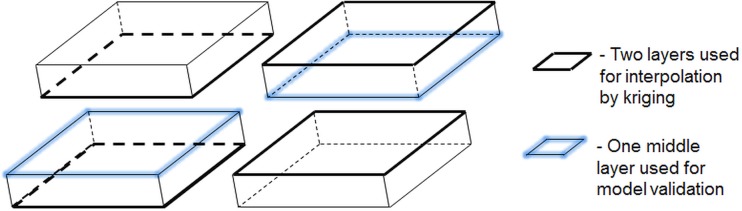
Illustration of two consecutive slices and the corresponding layers used in cross-validation. Schematic representation of two consecutive slices, for which chemical measurements on surface are available. From the two slices, three consecutive layers are used for model development and validation, upper and lower layers (thick black line) are used for model training and interpolation, and middle layer (blue shadowed line) is used for validation of model predictive performance.

The chemical elements used in the model, for which measurements were available, were silicon, carbon, iron and oxygen. The procedure for building and validating the statistical model was based on three consecutive layers from the 10 faces scanned by SEM-EDX. Of these three layers, two were faces of the same slice, and the third layer was one of the faces of the previous/next slice (Fig 3). Of the three consecutive layers, chemical and physical data on the upper and lower layers, positioned 65 voxel unit apart, were used to build the model, which was further used to predict the chemical structure of the internal 3D domain situated within these two layers. Validation of model predictive performance was done based on the middle layer for which measurements of the chemical elements were available (Fig 3). Given that each slice was approximately 250 μm thick (corresponding to 15 X-ray CT layers gap in the 3D X-ray CT image stack) and the distance between slices was approximately 790 μm (corresponding to 50 layers gap in the X-ray CT image stack, material that was lost during the cutting process), the middle layer, used for validation was not equally distant to the upper and lower layers, but it was closer to one of the two, depending on which three consecutive faces were chosen. This choice of three consecutive layers gave in total eight sets of three layer image data, which were used during the validation process, which was implemented as an eight-fold cross-validation (use two layers leave middle one out). To evaluate the predictive performance of the statistical model the Root Mean Square Error (RMSE) and the coefficient of determination (R^2^) were calculated based on the observed *O*_*i*_ and predicted values *P*_*i*_ using the following formulae:

$$RMSE\;=\;\sqrt{\;\sum {\left(P_{i}-O_{i}\right)}^{2}/N}\;and\;R^{2}\;=\;1-\sum {\left(O_{i}-P_{i}\right)}^{2}/\sum {\left(O_{i}-mean(O_{i})\right)}^{2},$$

where, *N* = 750 × 800 is the total number of voxels on the layer on which model validation was performed. We recognized that these two criteria would necessarily select the same predictive model from cross validation on a common data set. However, we computed and present both because the RMSE allows us to evaluate the prediction quality on the units of the original measurement, whereas the coefficient of determination presents a measure of predictive quality on a dimensionless bounded scale which can be compared between variables. Paired samples t-test was used to determine significant differences in means between these predictive performance measures as provided by regression tree and regression tree kriging. Likewise, paired sample t-test was used to compare predicted values (by each of the two methods) against observed values in terms of means and standard deviation.

## Results

### 3.1 Alignment of the 2D SEM-EDX chemical maps with the 3D X-ray CT volume

**Fig 4:**
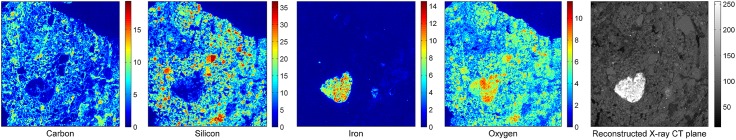
Illustration of SEM-EDX chemical mapping and the corresponding layer within the 3D X-ray CT data. The intensity of the colour indicates the concentration of the elements, whereas the grayscale intensity of the X-ray CT image reflects the density of the material.

The automated method developed previously by Hapca et al. [33] was successful in aligning the chemical maps within the 3D X-ray volume, as evinced by the satisfactory precision with which the localization of the corresponding 2D plane could be achieved. Good agreement was found in the estimation of the rotation parameters for the ten faces of the 5 slices. Rotation parameters *a* and *b* for alignment were consistently estimated to be 0.001 ± 0.002 and 0.033 ± 0.004, which shows that the five slices were cut almost parallel to each other. As can be noticed in the illustrative chemical maps in Fig 4, some experimental errors occurred during the cutting process with some of the slices being slightly or more severely broken. In terms of the distribution of the chemical components, it appears that carbon is mainly located within the pore space corresponding to the impregnated resin, silicon is found on the soil aggregate corresponding to intermediate X-ray grayscale intensities, iron correlates very well with high X-ray grayscale values, whereas oxygen is found everywhere in varying concentrations, except in the resin-impregnated pore space where it was consistently low.

### 3.2 Modelling the variogram and selection of the optimum sampling grid for regression kriging

**Fig 5:**
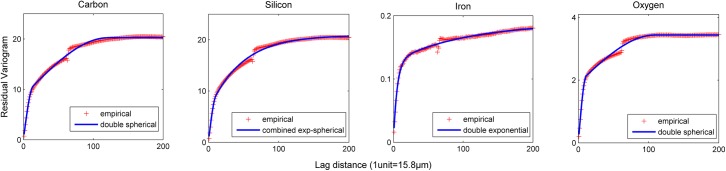
Variogram model fit for the residuals resulting from the regression tree model.

Among the different variogram models tested, in all replicates and for all four chemicals, the best fit as indicated by the AIC was provided by a four-parameter model corresponding either to the double spherical, or double exponential, or combined exponential-spherical with zero nugget (Fig 5).

**Fig 6:**
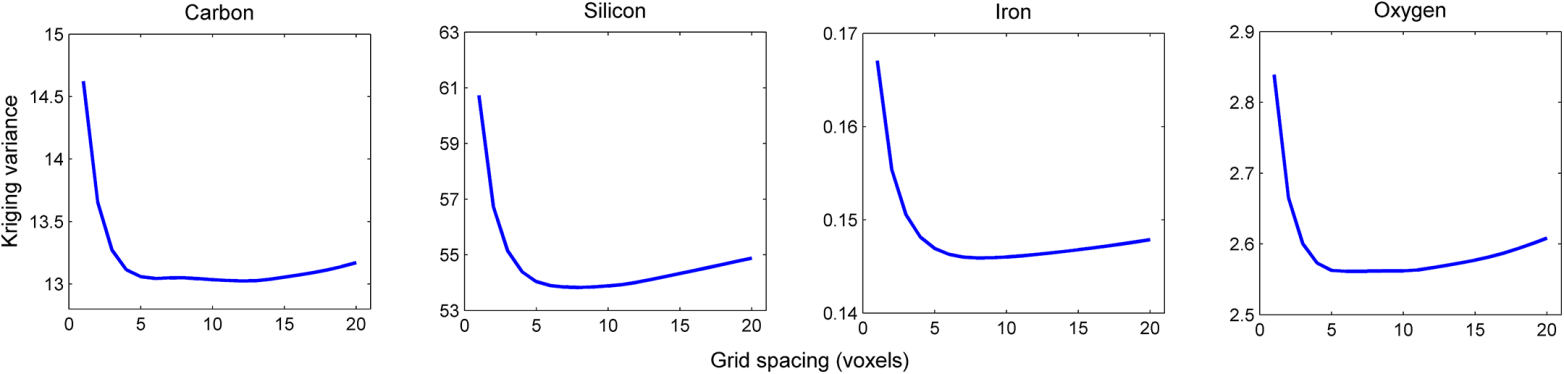
Method of selecting the optimum sampling grid based on the analysis of kriging variance. Representative profile of the kriging variance for a 9 × 9 sampling grid with grid spacing ranging from 1 to 20, when the target point is half way between the two interpolation layers.

To determine an optimum sampling for kriging, a 9 × 9 sampling grid with grid spacing ranging from 1 to 20 was tested. With a total of 2 × 81 = 162 kriging points sampled from both interpolation layers, a 9 × 9 sampling grid was considered a good trade-off between computational efficiency and representativeness of the sampling space for kriging. Plots of the kriging variance for different values of the grid space when the target point is half way between the two interpolation layers (i.e. at 32 voxel units distance from each of the two layers) indicates that the kriging variance becomes minimal for grid spacing between 5 and 10 voxel units (Fig 6). At smaller spacing, there is too much redundancy between the kriging points, whereas coarser spacing leads to an increased distance from the kriging points to the target point, and so results in less dependence. The profile of the kriging variance for the different values of the grid space was very similar for all four chemicals and results were consistent throughout all eight replicates. These kriging variance profiles suggest that a single grid spacing of 8 voxel units appears to be an appropriate value for the sampling grid to be used for all four chemicals.

### 3.3 Prediction of the 3D chemical structure: model assessment and comparison

**Fig 7:**
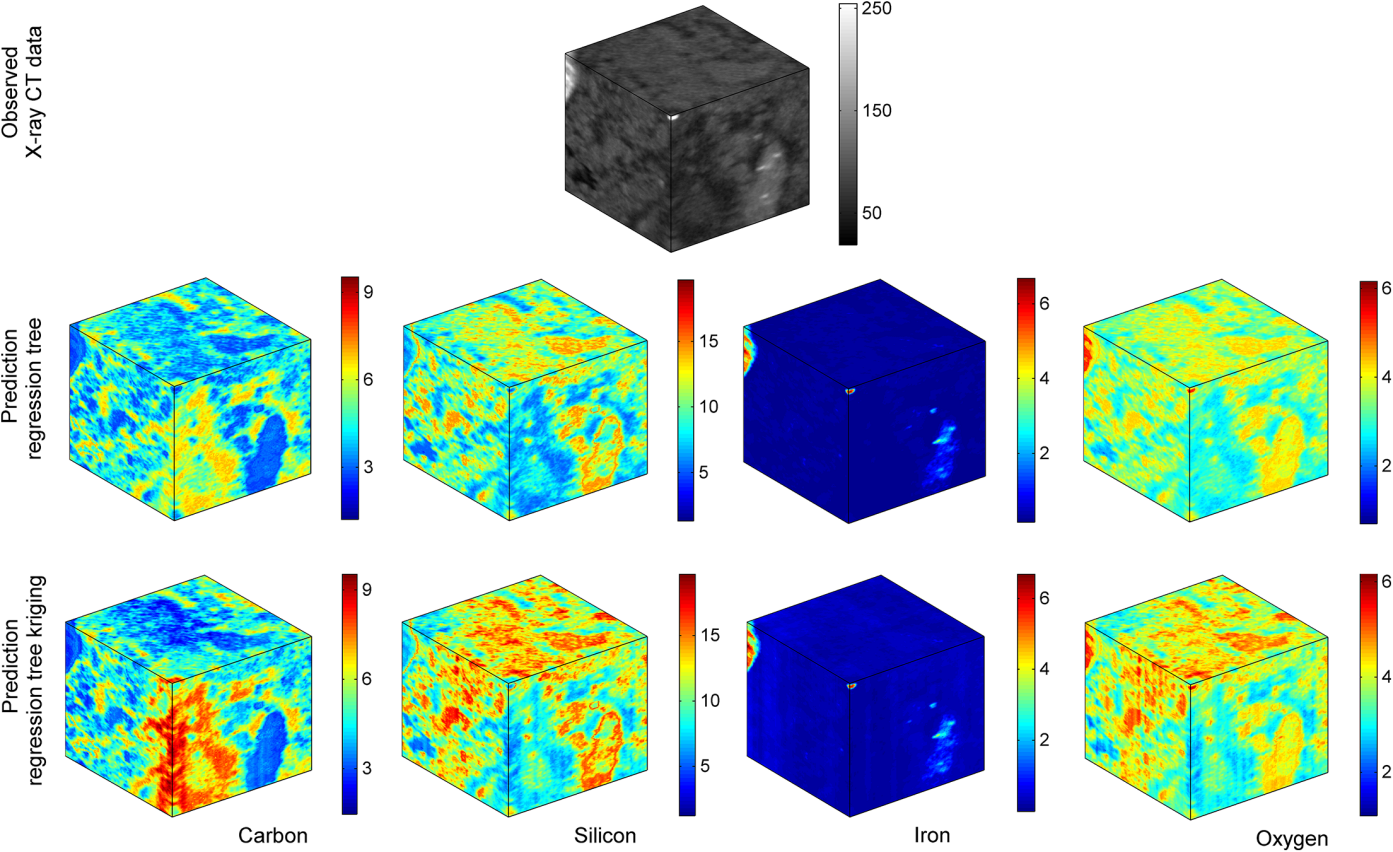
3D prediction of the chemical elements. Predicted 3D chemical structure by regression tree and regression tree kriging for a 128^3^ voxels domain and the corresponding 3D X-ray CT measurements.

The 3D chemical structure for a 128^3^ inner domain predicted by regression tree and regression-tree kriging, together with the 3D X-ray CT measurements correlates closely with the measured X-ray CT data, with carbon present in the pore space, silicon and oxygen on the solid phase and iron corresponding to large X-ray greyscale intensities (Fig 7).

**Fig 8:**
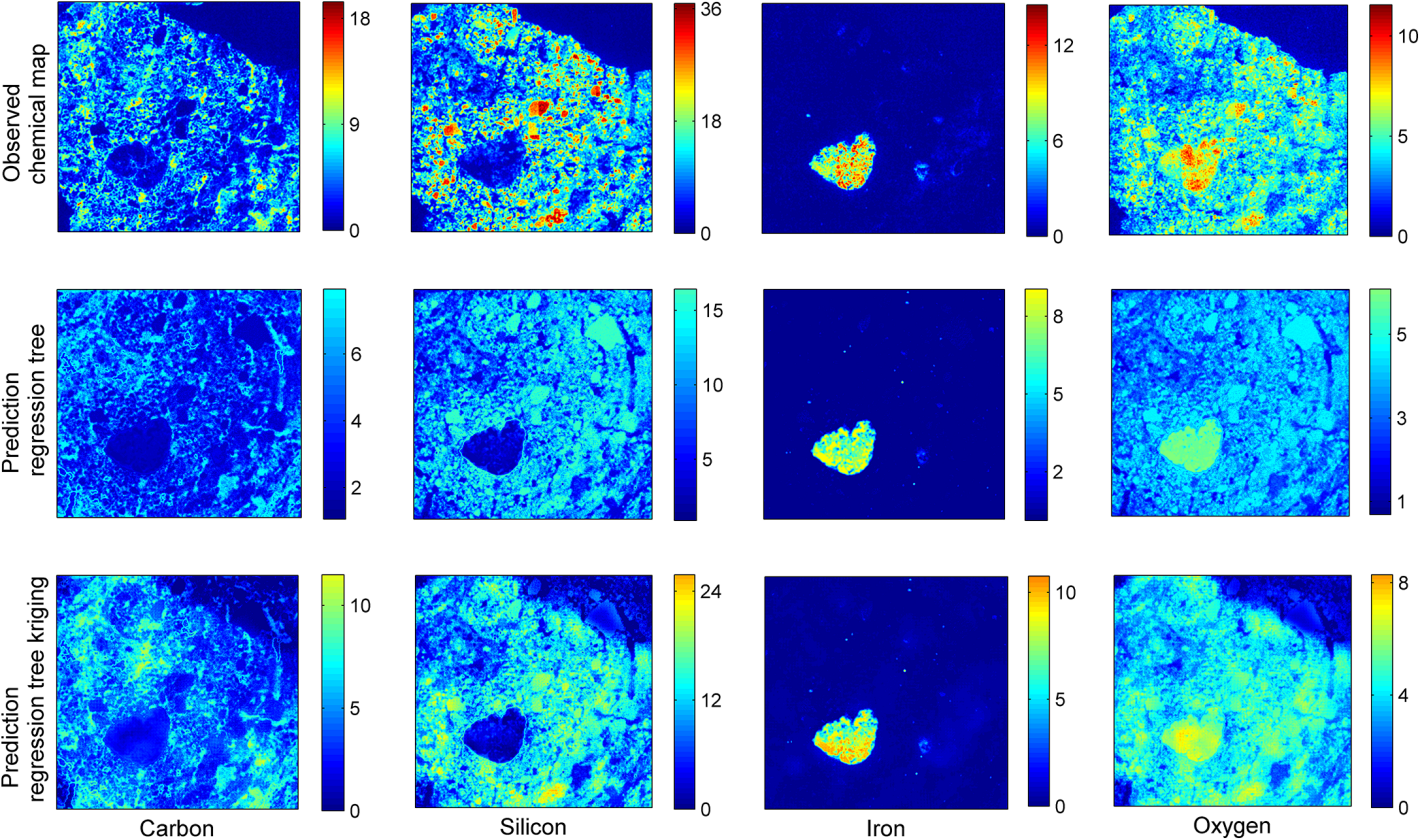
Evaluation of predictive performance. Prediction of the chemical composition of soil on a 2D layer by regression tree and regression tree kriging models, compared to actual measurements.

In general, both regression tree and regression-tree kriging methods succeeded in predicting correctly the location of chemicals (Fig 8). For both regression tree and regression-tree kriging method, mean predicated values in Table 1 are not significantly different from mean observed values (p-values>0.850). However, the standard deviation of the predicted values is significantly underestimated by both regression tree and regression-tree kriging, compared to the standard deviation of the measured chemical values. There is significantly more variation in the distribution of the chemical concentration as measured by the SEM-EDX method than that predicted by regression tree for all four chemicals (p-values<0.001 for carbon, silicon and oxygen and p-value<0.05 for iron). The additional kriging of the residuals induces more variation in the predicted values, but their standard deviation is still significantly underestimated for carbon, silicon and oxygen (p-values<0.001), whereas for iron the results are no longer significant (p-value = 0.153).

**Table 1:** Summary statistics of chemical values predicted by regression tree and regression tree kriging compared against observed values. Numbers show Mean and Standard Deviation *mean ± SE* over n = 8 replicates, on which the models were tested.

Chemical element	Error measure	Regression tree	Regression tree kriging	Observed Values
Carbon	Mean	5.333 ± 0.410	5.331 ± 0.416	5.351 ± 0.479
	SD	1.562 ± 0.096	2.188 ± 0.195	2.961 ± 0.206
Silicon	Mean	10.536 ± 0.356	10.531 ± 0.423	10.497 ± 0.528
	SD	3.880 ± 0.263	4.952 ± 0.276	6.627 ± 0.292
Iron	Mean	0.333 ± 0.041	0.332 ± 0.043	0.333 ± 0.045
	SD	0.620 ± 0.168	0.671 ± 0.170	0.727 ± 0.164
Oxygen	Mean	3.769 ± 0.159	3.763 ± 0.164	3.766 ± 0.188
	SD	0.847 ± 0.055	1.227 ± 0.079	1.589 ±0.104

Based on the goodness of fit measures (Table 2), both regression tree and regression tree-kriging provide a relatively good prediction of iron. Due to its high density, the X-ray grayscale intensities corresponding to iron are very large, and therefore, for some of the 2D layers the regression tree model could explain as much as 90% of the variability in the data (i.e., R^2^>0.90). The fact that the distribution of iron in the soil sample is relatively sparse also made it easier to locate it within the 3D volume. However, for other chemicals, although prediction of their location by regression tree was generally correct, the large concentration values were under-predicted by this method alone (Fig 8). Additional kriging of the residuals improved significantly the prediction performance of the regression tree model as indicated by the significant decrease in the RMSE for all four chemicals (p-values<0.05). This is particularly the case for carbon, silicon and oxygen, the spatial distribution of which is more homogeneous within the soil structure. For these chemical elements it appears that the spatial correlation in the residual structure represents an important component to the model, with a significant increase in the R^2^ value from 0.221 to 0.324 for predicted carbon, 0.312 to 0.423 for silicon, and 0.218 to 0.374 for predicted oxygen, whereas for iron the R^2^ value has increased from 0.525 to 0.583 (Table 2).

**Table 2:** Comparison of predictive performance by regression tree and regression tree kriging. Numbers show RMSE and R^2^ *mean ± SE* over n = 8 replicates, on which the models were tested.

Chemical element	Error measure	Regression tree	Regression tree kriging
Carbon	RMSE	2.617 ± 0.203	2.418 ± 0149
	R^2^	0.221 ± 0.031	0.324 ± 0.034
Silicon	RMSE	5.474 ± 0.231	4.990 ± 0.172
	R^2^	0.312 ± 0.031	0.423 ± 0.032
Iron	RMSE	0.369 ± 0.030	0.349 ± 0.030
	R^2^	0.525 ± 0.126	0.583± 0.117
Oxygen	RMSE	1.398 ± 0.083	1.229 ± 0.042
	R^2^	0.218 ± 0.039	0.374± 0.049

## Discussion and Conclusions

There is increasing evidence that quantitative characterization of the soil micro-environment and of its heterogeneity holds the key to a more precise prediction of soil ecosystem functioning [13, 42–44]. Despite tremendous progress over recent years, techniques to quantify the physical, biological and chemical characteristics of soil have developed within separate disciplines and operate at different spatial scales, ranging from non-invasive 3-dimensional to destructive 2-dimensional methods. The present study breaks new ground in that it addresses these challenges by developing an integrated experimental and theoretical framework based on a spatial statistics approach that combines X-ray CT imaging technique for the 3D characterisation of the soil structure with SEM-EDX 2D chemical maps in order to predict the 3D chemical composition of the inner soil.

As part of the proposed methodology, an automated statistical method developed by Hapca et al. [33] was first applied to align the 2D SEM-EDX chemical maps within the 3D X-ray CT physical volume. A regression tree method was then developed to predict the 3D chemical structure based on the 3D X-ray CT data. Due to the strong correlation between the chemical and the physical structure, the regression tree based method was in general successful in predicting the location of the chemicals and the overall mean concentration. However, high concentrations of measured chemicals were generally underestimated by this method and this was reflected in a significantly lower variability in the predicted values, compared to the variability of the actual measurements. Additional interpolation of the regression tree residuals by Ordinary Kriging increased significantly the variability in the predicted chemical values, improving at the same time the predictive performance of the model in terms of both predictive-error RMSE and goodness-of-fit R^2^.

Due to the large amount of data available, implementation of Ordinary Kriging to imaged data requires the design of a sampling scheme for the interpolation points to be used in kriging. In particular, due to a series of limitation in the slicing process, the 2D chemical planes were quite distant from each other and the classical approach of using contiguous subsets of voxels in the prediction layers did not give optimum results. To overcome this, we developed a methodology for optimizing the choice of the sampling grid based on the minimization of the kriging variance. The proposed sampling procedure can be generally used when kriging methods are to be applied to imaged data.

A regression tree approach was found suitable to model the relationship between the X-ray CT grayscale data and the SEM-EDX chemical maps. This is likely due to the fact that the X-ray CT imaging process consists of measurements of the attenuation of X-rays passing through given materials. As a result, each grayscale value in an X-ray CT volume file represents the average density of the corresponding voxel through which X-rays are attenuated, which in turn depends on the chemical composition of the material [36, 45]. Therefore, at least theoretically, the chemical composition of the material can be mathematically mapped into the X-ray CT grayscale values by means of a step function. However, the step-function-based regression tree model is a non-linear approach where one can fit as many parameters as one wants and, therefore, pruning the tree in order to limit the number of parameters is an essential step to avoid over-fitting the data. In this sense, more complex linear models such as regression splines models [40] should be further explored in order to improve the 3D predictive performance. On the other hand, in this study, the additional kriging of the residuals was based on a very simplistic assumption of isotropy of the spatial data. However, previous evidence has revealed that soils are very heterogeneous in their physical and chemical structure, showing different characteristics when the direction of measurements is changed [46–48]. As a result, the isotropy might not be a realistic assumption for the soil data, and fitting anisotropic models to the experimental variogram should be further explored.

In general, the process of data collection is bound to cause experimental errors. For the present study, a major difficulty consisted of cutting fine slices without breaking the materials. This has been only partially successful here, several of the slices ending up slightly or more severely broken during the cutting process (Fig 7, top layer images). This affected the alignment process as well as the goodness of fit of predicted values to the observed data. Moreover, the quantity of soil material wasted during the cutting process was almost three times thicker than the slices themselves, so that the succession of surfaces scanned did not occur at equal distances. This relative lack of control over the spacing between slices might have had an effect on the validation results. An alternative to slicing, used previously by Eickhorst, and Tippkötter [19] is to ground down the surface of the soil sample as much as required after each SEM-EDX scanning; in this way the distance at which one scan each 2D surface by SEM-EDX can be better planned. In addition to standard experimental errors, the imaging process in itself poses another set of challenges. In particular, for soil imaging, the irregular distribution of soil minerals, unique to each sample, may lead to different levels of noise across the resulting images. Also, the complex spatial structure of soils, exceeding the resolution of the imaging system, produces a partial volume effect in the reconstructed image, with a large proportion of image elements consisting of a mixture of pore and solid material phases, which produces an overlap in grayscale values corresponding to materials of different chemical components [9,35,36,45]. As a result the quality of the X-ray images plays an important role in the predictive modelling process of the 3D chemical structure.

Prediction of the 3D chemical composition was done in this first study only for carbon, silicon, iron and oxygen. Although other chemicals were initially scanned by SEM-EDX, such as titanium and potassium, these elements were present in the soil only in small quantities. It was difficult to correlate the chemical maps of these elements with the X-ray CT data, and therefore it was decided not to use them for the analysis. On the other hand, a key aspect of the experimental framework consists of resin impregnation of the soil sample. During this process the soil pore space gets filled with resin and the resulting SEM-EDX carbon map correlates well with the corresponding pore space in the X-ray CT data. Although the SEM-EDX technology allows differentiation between the carbon naturally occurring in soil and the carbon in the resin based on O:C values, the 3D X-ray scans are still limited in this respect. Quantification/segmentation of the soil carbon from the X-ray CT image data represents at the moment a real challenge [49,50], and therefore image analysts might be able to advance with respect to this topic by bridging the X-ray CT imaging with the SEM-EDX technology for the development/validation of appropriate multi-phase segmentation methods to help locate carbon in 3D micro-soil images.

The present study is the first of its kind to produce 3D maps of chemical elements at the microscale, on the basis of 2D maps established on cuts through a soil sample. Although further adjustments of the experimental and theoretical framework can help improve model predictive performance, the early stage of the work presented here represents a significant advance in soil research, showcasing how integration of physical and chemical techniques combined with modelling can help predict the 3D chemical elements in soil. The methodology presented can be further adapted for prediction of the 3D biological data, which together with the physical and chemical structure can help advance our understanding of the 3D soil micro-environment.

## References

[1] Alexander M. Biochemical ecology of soil microorganisms. Annu Rev Microbiol. 1964; 18:217–250. 10.1146/annurev.mi.18.100164.001245 14268858

[2] Elliot TR, Heck RJ. A comparison of optical and X-ray CT technique for void analysis in soil thin section. Geoderma. 2007; 141:60–70. 10.1016/j.geoderma.2007.05.001

[3] Taina IA, Heck RJ, Elliot TR. Application of X-ray computed tomography to soil science: A literature review. Can J Soil Sci. 2008; 88:1–20. 10.4141/CJSS06027

[4] Iassonov P, Gebrenegus T, Tuller M. Segmentation of X-ray computed tomography images of porous materials: A crucial step for characterization and quantitative analysis of pore structures. Water Resour Res. 2009; 45:W09415 10.1029/2009WR008087

[5] Long HL, Swennen R, Foubert A, Dierick M, Jacobs P. 3D quantification of mineral components and porosity distribution in Westphalian C sandstone by microfocus X-ray computed tomography. Sediment Geol. 2009; 220:116–125. 10.1016/j.sedgeo.2009.07.003

[6] Yao YB, Liu DM, Che Y, Tang DZ, Tang SH, Huang WH. Non-destructive characterization of coal samples from China using microfocus X-ray computed tomography. Int J Coal Geol. 2009; 80:113–123. 10.1016/j.coal.2009.08.001

[7] Iassonov P, Tuller M. Application of segmentation for correction of intensity bias in x-ray computed tomography images. VZJ. 2010; 9(1):187–191. 10.2136/vzj2009.0042

[8] Baveye PC, Laba M, Otten W, Bouckaert L, Dello Sterpaio P, Goswami RR, et al Observer-dependent variability of the thresholding step in the quantitative analysis of soil images and X-ray microtomography data. Geoderma. 2010; 157:51–63. 10.1016/j.geoderma.2010.03.015

[9] Hapca SM, Houston A, Otten W, Baveye PC. New local thresholding method for soil images by minimizing grayscale intra-class variance. VZJ. 2013; 12(3).

[10] Schumacher M, Christl I, Scheinost AC, Jacobsen C, Kretzschmar R. Chemical heterogeneity of organic soil colloids investigated by scanning transmission X-ray microscopy and C-1s NEXAFS microspectroscopy. Environ Sci Technol. 2005; 39(23):9094–9100. 10.1021/es050099f 16382929

[11] Wan J, Tyliszczak T, Tokunaga TK. Organic carbon distribution, speciation, and elemental correlations within soil micro aggregates: Applications of STXM and NEXAFS spectroscopy. Geochim Cosmochi Ac. 2007; 71:5439–5449. 10.1016/j.gca.2007.07.030

[12] Solomon D, Lehmann J, Kinyangi J, Liang BQ, Heymann K, Dathe L, et al Carbon (1s) NEXAFS Spectroscopy of biogeochemically relevant reference organic compounds. Soil Sci Soc Am J. 2009; 73:1817–1830. 10.2136/sssaj2008.0228

[13] Jacobson AR, Dousset S, Andreux F, Baveye PC. Electron microprobe and synchrotron X-ray fluorescence mapping of the heterogeneous distribution of copper in high-copper vineyard soils. Environ Sci Technol. 2007; 41:6343–6349. 10.1021/es070707m 17948777

[14] Strawn DG, Baker LL. Speciation of Cu in a contaminated agricultural soil measured by XAFS, mu-XAFS, and mu-XRF. Environ Sci Technol. 2008; 42:37–42. 10.1021/es071605z 18350872

[15] Strawn DG, Baker LL. Molecular characterization of copper in soils using X-ray absorption spectroscopy. Environ Pollut. 2009; 157:2813–2821. 10.1016/j.envpol.2009.04.018 19446385

[16] Prietzel J, Thieme J, Salome M. Assessment of sulfur and iron speciation in a soil aggregate by combined S and Fe micro-XANES: microspatial patterns and relationships. J Synchrotron Radiat. 2010; 17:166–172. 10.1107/S0909049509049917 20157267

[17] Thieme J, Sedlmair J, Gleber SC, Prietzel J, Coates J, Eusterhues K, Abbt-Braun G, Salome M. X-ray spectromicroscopy in soil and environmental sciences. J Synchrotron Radiat. 2010; 17:149–157. 10.1107/S0909049509049905 20157265

[18] Eickhorst T, Tippkötter R. Detection of FISH-stained microorganisms in soil microstructure by fluorescence microscopy. Microsc Microanal. 2008; 14:754–755. 10.1017/S1431927608083025

[19] Eickhorst T, Tippkötter R. Detection of microorganisms in undisturbed soil by combining fluorescence in situ hybridization (FISH) and micropedological methods. Soil Biol Biochem. 2008; 40:1284–1293. 10.1016/j.soilbio.2007.06.019

[20] Clode PL, Kilburn MR, Jones DL, Stockdale EA, Cliff JB, Herrmann AM, et al In situ mapping of nutrient uptake in the rhizosphere using nanoscale secondary ion mass spectrometry. Plant Physiol. 2009; 151:1751–1757. 10.1104/pp.109.141499 19812187 PMC2785960

[21] DeLeo PC, Baveye P, Ghiorse WC. Use of confocal laser scanning microscopy on soil thin sections for improved characterization of microbial growth in unconsolidated soils and aquifer materials. J Microbiol Meth. 1997; 30(3):193–203. 10.1016/S0167-7012(97)00065-1

[22] Moreau E, Velde B, Terribile F. Comparison of 2D and 3D images of fractures in a Vertisol. Geoderma. 1999; 92:55–72. 10.1016/S0016-7061(99)00025-7

[23] Van Geet M., Swennen R., Wevers M., 2000 Quantitative analysis of reservoir rocks by microfocus X-ray computerised tomography. Sedimentary Geology 132, 25–36.

[24] Okabe H, Blunt MJ. Prediction of permeability for porous media reconstructed using multiple-point statistics. Phys Rev E. 2004; 70:066135 10.1103/PhysRevE.70.066135 15697462

[25] Remeysen K, Swennen R. Application of microfocus computed tomography in carbonate reservoir characterization: Possibilities and limitations. Mar Petrol Geol. 2008; 25:486–499. 10.1016/j.marpetgeo.2007.07.008

[26] Thullner M, Baveye P. Computational pore network modeling of the influence of biofilm permeability on bioclogging in porous media. Biotech Bioeng. 2008; 99(6):1337–1351. 10.1002/bit.21708 18023059

[27] Prado B, Duwig C, Marquez J, Delmas P, Morales P, James J, et al Image processing-based study of soil porosity and its effect on water movement through Andosol intact columns. Agr Water Manag. 2009; 96:1377–1386. 10.1016/j.agwat.2009.04.012

[28] Pajor R, Falconer R, Hapca S, Otten W. Modelling and quantifying the effect of heterogeneity in soil physical conditions on fungal growth. Biogeosci. 2010; 7:3731–3740. 10.5194/bg-7-3731-2010

[29] Rau C, Somogyi A, Simionovici A. Microimaging and tomography with chemical speciation. Nucl Instrum Methods B. 2003; 200:444–450. 10.1016/S0168-583X(02)01737-8

[30] Bleuet P, Lemelle L, Tucoulou R, Gergaud P, Delette G, Cloetens P. et al 3D chemical imaging based on a third-generation synchrotron source. Trend Anal Chem. 2010; 29(6):518–527. 10.1016/j.trac.2010.02.011

[31] Willson CS, Lu N, Likos WJ. Quantification of Grain, Pore, and Fluid Microstructure of Unsaturated Sand from X-Ray Computed Tomography Images. Geotech Test J. 2012; 35 (6):1–13. 10.1520/GTJ20120075

[32] Shokri N. Pore-scale dynamics of salt transport and distribution in drying porous media. Phys Fluids. 2014; 26:012106 10.1063/1.4861755

[33] Hapca SM, Wang ZX, Otten W, Wilson C, Baveye PC. Automated statistical method to align 2D chemical maps with 3D X-ray computed micro-tomographic images of soils. Geoderma. 2011; 164:146–154. 10.1016/j.geoderma.2011.05.018

[34] Wilson CA, Davidson DA, Cresser MS. Multi-element soil analysis: an assessment of its potential as an aid to archaeological interpretation. J Archaeol Sci. 2008; 35 (2):412–424. 10.1016/j.jas.2007.04.006

[35] Houston A, Baveye P, Otten W, Hapca S. Adaptive-window indicator kriging: A thresholding method for computed tomography images of porous media. Comput Geosci. 2013; 54:239–248. 10.1016/j.cageo.2012.11.016

[36] Houston AN, Schmidt S, Tarquis AM, Otten W, Baveye PC, Hapca SM. Effect of scanning and image reconstruction settings in X-ray computed microtomography on quality and segmentation of 3D soil images. Geoderma. 2013; 207-208(1):154–165. 10.1016/j.geoderma.2013.05.017

[37] Stein ML. Interpolation of Spatial Data: Some theory for kriging. Springer, New York; 1999 pp. 247.

[38] Lark RM, Cullis BR, Welham SJ. On spatial prediction of soil properties in the presence of a spatial trend: the empirical best linear unbiased predictor (E-BLUP) with REML. Eur J Soil Sci. 2006; 57:787–799. 10.1111/j.1365-2389.2005.00768.x

[39] Hengl T, Heuvelink GMB, Stein A. A generic framework for spatial prediction of soil variables based on regression-kriging. Geoderma, 2004; 122(1–2):75–93. 10.1016/j.geoderma.2003.08.018

[40] Hastie T, Tibshirani R, Friedman J. Elements of statistical learning: Data mining, Inference and Prediction, 2nd edn., Springer; 2009 pp. 552.

[41] Webster R, Oliver MA. Geostatistics for environmental scientists John Wiley & Sons Inc.; 2007 pp 315.

[42] Otten W, Harris K, Young IM, Ritz K, Gilligan CA. Preferential spread of the pathogenic fungus Rhizoctonia solani through structured soil. Soil Biol Biochem. 2004; 36:203–210. 10.1016/j.soilbio.2003.09.006

[43] Kravchenko A, Falconer R, Grinev D, and Otten W. Fungal colonization in soils of contrasting managements: modelling fungal growth in 3D pore volumes of undisturbed soil samples. Ecol Appl. 2011; 21(4):1202–1210. 10.1890/10-0525.1 21774424

[44] Cazelles K, Otten W, Baveye PC, Falconer RE. Soil fungal dynamics: Parameterization and sensitivity analysis of modelled physiological processes, soil architecture and carbon distribution. Ecol Model. 2013; 248:165–173. 10.1016/j.ecolmodel.2012.08.008

[45] Sleutel S, Cnudde V, Masschaele B, Vlassenbroek J, Dierick M, Van Hoorebeke L, et al Comparison of different nano- and micro-focus X-ray computed tomography set-ups for the visualization of the soil microstructure and soil organic matter. Comput Geosci. 2008; 34(8):931–938. 10.1016/j.cageo.2007.10.006

[46] Garnier P, Perrier E, Jaramillo RA, Baveye P. Numerical model of 3-dimensional anisotropic deformation and 1-dimensional water flow in swelling soils. Soil Sci. 1997; 162:410–420. 10.1097/00010694-199706000-00003

[47] Peng X, Horn R. Anisotropic shrinkage and swelling of some organic and inorganic soils. Eur J Soil Sci. 2007; 58:98–107. 10.1111/j.1365-2389.2006.00808.x

[48] Gebhardt S, Fleige H, Horn R. Anisotropic shrinkage of mineral and organic soils and its impact on soil hydraulic properties. Soil Till Res. 2012; 125:96–104. 10.1016/j.still.2012.06.017

[49] Schlüter S, Sheppard A, Brown K, Wildenschild D. Image processing of multiphase images obtained via X-ray microtomography: A review. Water Resour Res. 2014; 50(4):3615–3639. 10.1002/2014WR015256

[50] Van Loo D, Bouckaert L, Leroux O, Pauwels E, Dierick M, Van Hoorebeke L, et al Contrast agents for soil investigation with X-ray computed tomography. Geoderma. 2014; 213:485–491. 10.1016/j.geoderma.2013.08.036

